# Supplementary material for: Cohort profile: Data standards for cardiac rehabilitation structure and processes for the SWEDEHEART cardiac rehabilitation (SWEDEHEART-CR) registry
Source: PLoS One. 2023 Nov 3;18(11):e0293840. doi: 10.1371/journal.pone.0293840 (PMC10624275; doi:10.1371/journal.pone.0293840)
Supplement: S1 Table — (PDF) [file pone.0293840.s002.pdf]

S1 Table. A list of candidate variables.

Swedish (original language)	English translation
PATIENTER	PATIENTS
Alla patienter med genomgånget AKS (hjärtinfarkt eller instabil angina) och/eller revaskularisering (CABG eller PCI) erbjuds deltagande i centerbaserat hjärtrehabiliteringsprogram hos oss.	We offer participation in centre-based cardiac rehabilitation (CR) to all patients hospitalised for acute coronary syndrome (myocardial infarction or unstable angina) and/or revascularization (CABG or PCI).
Även patienter med kranskärlssjukdom, utan föregående AKS eller revaskularisering, erbjuds tillgång till hjärtrehabiliteringsprogram hos oss inom första året efter att diagnos har satts.	Even patients with chronic coronary syndrome, without previous ACS or revascularization, are offered participation in a CR programme within the first year after initial diagnosis.
ORGANISATION OCH PERSONAL	ORGANISATION AND HUMAN RESOURCES
Programmet startar tidigt efter utskrivning, företrädesvis inom 1–2 veckor och senast 4 veckor efter utskrivning.	The CR programme starts early after discharge, preferably within 1-2 weeks, and within 4 weeks after discharge at the latest. *
Programmet varar minst 3–6 månader.	The duration of the CR programme is at least 3-6 months.
Vi erbjuder strukturerat och multiprofessionellt omhändertagande, där läkare, sjuksköterskor och fysioterapeuter, samt övriga professioner med viktiga stödfunktioner (i.e. psykologer/kuratorer, tobaksavvänjare och dietister) arbetar tillsammans med patienterna utifrån ett personcentrerat förhållningssätt, baserat på det individuella behovet.	We provide structured care by a multiprofessional team including physicians, nurses, physiotherapists, and other supporting professions (i.e., psychologists, social workers, tobacco counsellors and dieticians) who work together with the patient in a person-centred manner, based on the patient´s individual needs.
Vår hjärtrehabiliteringsenhet har en medicinskt ansvarig läkare.	Our CR centre has a medical director.
Personal som arbetar inom hjärtrehabilitering hos oss har utbildning i samtalsmetodik, exempelvis motiverande samtalsmetodik.	Personnel at our CR centre has training in counselling methods (e.g., motivational interviewing or cognitive behavioural therapy).
Minst en i teamet har utbildning i kvalificerad tobaksavvänjning.	At least one member of our CR team has training in tobacco counselling.
Samtliga professioner som har patientnära arbete hos oss är utbildade i S-HLR.	All individuals in our CR team who have direct patient contact have training in CPR.
Våra sjuksköterskor har individuell behörighet (i.e. delegering) att justera doseringen av lipidsänkande läkemedel samt läkemedel för högt blodtryck och hjärtsvikt.	Nurses at our CR centre have an individual delegation to titrate dosage/suggest changes in lipid lowering and blood pressure lowering medication.
Vi har regelbundna schemalagda ronder, där enskilda patientärenden diskuteras i teamet.	We have regular interdisciplinary team meetings to discuss patient cases.
Vi har regelbundna team-möten, för att till exempel diskutera arbetsfördelning, behandlingskvalitet och förbättringsarbete samt för att förstärka team-arbetet.	We have regular interdisciplinary team meetings to discuss operational matters such as work routines, quality of care, and to improve the team spirit.

Vi rapporterar till det svenska sekundärpreventiva kvalitetsregistret SEPHIA.	We report data to the SWEDEHEART-CR registry.**
Vi följer kontinuerligt våra SEPHIA-resultat och använder dessa för utveckling av verksamheten och kvalitetsförbättrande åtgärder.	We use SWEDEHEART-CR data continuously to follow and improve quality of care at our CR centre.
Vid första besök hos sjuksköterska och/eller läkare (inom 1–2 veckor) diskuteras individuella mål för riskfaktorer, levnadsvanor samt läkemedelsbehandling och de mest angelägna målen lyfts fram i samråd med patienten.	During the initial assessment with the nurse and/or physician (within 1-2 weeks), individual goals for risk factors, lifestyle habits, and pharmacological treatment are discussed, and the most important goals are highlighted in consultation with the patient.
Patienten ges skriftliga/digitala kopior på sina värden, läkemedelslista och vilka mål som bör eftersträvas, med en tydlig, tillsammans med patienten författad plan för hur detta kan göras.	The patient is provided with written/digital copies of their values, medication list, and the goals to be pursued, along with a clear plan co-authored with the patient on how this can be achieved.
Identifierade och förändringsbara riskfaktorer, målvärden, och läkemedelsbehandling följs upp.	We follow and act on the patient's identified modifiable risk factors and adherence to and effect of pharmacological treatment.
Vi erbjuder flexibilitet avseende kontaktform (fysiska besök, telefonkontakter, digitala kontakter).	We offer flexibility in terms of the mode of contact (physical visits, phone calls, digital contacts).
Vi strävar efter kontinuitet i vårdgivarkontakten under hela behandlingstiden.	We strive for continuity in patient-caretaker contact throughout follow-up.
Vi erbjuder närstående delta vid besök på hjärtrehabiliteringsenheten.	We offer the patient's relatives to attend follow-up visits.
För patienter som inte pratar svenska, används auktoriserad tolk.	For non-Swedish speaking patients, certified interpreter services are used.
LEVNADSVANOR	LIFESTYLE
Tobak	*Tobacco*
Vid första kontakt följs tobaksavvänjningsåtgärder, som initierats under vårdtiden, systematiskt upp.	At the initial assessment, smoking cessation measures initiated during hospitalization are systematically followed up. **
Dagligrökare erbjuds kvalificerat rådgivande samtal för att förändra sina tobaksvanor.	Daily smokers are offered structured counselling to change their tobacco habits.
Dagligsnusare erbjuds rådgivande samtal.	Daily users of wet snuff are offered counselling.
Vid behov remitteras patienter till diplomerad tobaksavvänjare inom primärvården eller till specialiserad tobaksavvänjningsenhet.	If necessary, patients are referred to a professional tobacco cessation counsellor in primary care or to a specialized tobacco cessation unit.
Nikotinersättningsmedel samt behandling med vareniklin eller bupropion erbjuds.	Nicotine-replacement therapy bupropion, cytisine, and/or varenicline therapy is offered to smokers.
Fysisk aktivitet	*Physical activity*
Patienter erhåller rekommendationer avseende ökad fysisk aktivitet och minskat stillasittande.	Patients receive recommendations for increased physical activity and reduced sedentary time. **
Varje patient får dessutom individuellt utformade råd om fysisk aktivitet av	Additionally, each patient receives individually tailored advice on physical activity from a

fysioterapeut efter att ha utvärderats avseende fysisk kapacitet.	physiotherapist after being evaluated for physical capacity.
Matvanor	*Dietary habits*
Kartläggning av matvanor ingår i det sekundärpreventiva arbetet hos oss.	Assessment of dietary habits is included in routine follow-up. **
Patienter som har ohälsosamma matvanor erbjuds kvalificerat rådgivande samtal för att förändra sina levnadsvanor. Vid behov konsulteras en dietist.	Patients with unhealthy dietary habits are offered structured counselling, with referral to a dietician if needed.
Alkoholvanor	*Alcohol habits*
Kartläggning av alkoholvanor ingår i det sekundärpreventiva arbetet hos oss.	Assessment of alcohol consumption is included in routine follow-up.
Rådgivande samtal ges till patienter som har ett riskbruk/skadligt bruk av alkohol. Vid behov remitteras patienterna till enheter med specialistkompetens.	Patients identified as having unhealthy use of alcohol are offered counselling, with referral to a specialist care if needed.
Vikt och midjemått	*Weight and weight circumference*
BMI används för att definiera kategorier av kroppsvikt (undervikt, normalvikt, övervikt och fetma). Mätning av midjemått används också.	BMI is used to define categories of body weight (underweight, normal weight, overweight, and obesity). Waist circumference is also measured.
Patienter med normalt BMI och midjemått uppmuntras till att behålla sin kroppsvikt, medan patienter med övervikt, fetma och/eller bukfetma uppmuntras till viktnedgång.	Patients with a normal BMI and waist circumference are encouraged to maintain their body weight, while patients with overweight, obesity, and/or abdominal obesity are encouraged to lose weight.
I vissa fall skickas remiss till dietist eller specialiserad överviktsenhet.	When indicated, a referral is made to a dietician or specialized weight management unit.
FYSISK TRÄNING INOM HJÄRTREHABILITERING	EXERCISE-BASED CARDIAC REHABILTAITON
Alla patienter erhåller bedömning med pre-exercise screening, aerob och muskulär kapacitet samt självrapporterad fysisk kapacitet och fysisk aktivitets- och träningsnivå hos en fysioterapeut.	All patients undergo a pre-exercise assessment visit with a physiotherapist. The pre-exercise assessment includes testing of aerobic and muscular capacity, assessment of self-reported physical capacity, physical activity, and exercise levels.
Första besök till fysioterapeut sker inom 1–2 veckor efter utskrivning (4 veckor om patienten genomgått CABG).	The pre-exercise assessment with a physiotherapist takes place within 1-2 weeks after discharge (4 weeks if the patient has undergone CABG). *
Individuellt anpassat träningsprogram förskrivs i samråd med patienten.	An individually tailored exercise programme is prescribed in consultation with the patient.
Träning påbörjas tidigast möjligt efter den inledande bedömningen, och utförs som fysioterapeutledd fysisk träning inom centerbaserad hjärtrehabilitering.	Exercise training is initiated as early as possible after the pre-exercise assessment and is conducted as physiotherapist-led exercise-based CR programme. *
Träningsprogrammet är minst 3 månader.	We offer participation in a supervised exercise-based CR programme for at least 3 months (24 sessions). *

Kompletterande individanpassad träning på måttlig till hög intensitetsnivå på egen hand uppmuntras.	Supplementary to participation in a supervised exercise-based CR programme, individually tailored exercise training at a moderate to high intensity level on one's own is encouraged.
Efter genomfört deltagande i fysisk träning utförs samma tester avseende fysisk kapacitet som vid det inledande besöket. Även patienter som inte har haft möjlighet att träna inom hjärtrehabilitering erbjuds denna uppföljande bedömning.	After completing an exercise-based CR programme, the same tests of aerobic and muscular capacity are conducted as during the pre-exercise assessment visit. This close-out visit is also offered to patients who have not had the opportunity to participate in an exercise-based CR programme at the CR centre. *
I samband med den avslutande bedömningen erbjuds patienten skriftlig ordination av fysisk aktivitet (Fysisk aktivitet på Recept, FaR).	During the close-out visit, the patient is offered a written prescription for physical activity.
Patienter som har fortsatt behov av handledd fysisk träning överförs till fysioterapeut i primärvården för fortsatt behandling.	Patients who have a continued need for supervised exercise training are referred to a physiotherapist in primary care for continued treatment.
Våra fysioterapeuter bistår med kunskapsstöd och rådgivning till fysioterapeuter i primärvården.	The physiotherapists at our CR centre provide knowledge support and guidance to physiotherapists in primary care.
LÄKEMEDEL	*MEDICATION*
Samtliga prognosförbättrande läkemedel sätts in hos patienter med kranskärlssjukdom tidigast möjligt efter att diagnosen har satts.	Secondary preventive medication is initiated as early as possible after the diagnosis of coronary artery disease has been established.
Om målvärden för LDL-kolesterol, blodtryck och blodsocker inte uppnås intensifieras behandlingen utan onödigt lång fördröjningstid.	If target levels for LDL-C, blood pressure, and blood sugar are not achieved, treatment is intensified without unnecessary delay.
Vi för en personcentrerad dialog med patienten kring läkemedel, där bland annat indikationer, behandlingslängd och vikten av följsamhet diskuteras.	We conduct a person-centred dialogue regarding medication with the patient, discussing aspects such as indications, duration of treatment, and the importance of adherence.
BLODTRYCK	*BLOOD PRESSURE*
Blodtryck kontrolleras vid varje besök hos sjuksköterska, läkare och vid behov hos fysioterapeut.	Blood pressure is measured at all follow-up visits with nurses and physicians, and as needed at follow-up visits with physiotherapists. **
För patienter som har högt blodtryck vid kontroll på mottagningen görs kompletterande hem-blodtryck och/eller 24-timmars blodtrycksmätning.	For patients with high office blood pressure, we measure home and/or ambulatory blood pressure.
LIPIDER	*LIPIDS*
Lipider kontrolleras inom 1–2 månader från utskrivning. Om målen inte uppnås med statin i högsta tolerabla dos, övervägs tillägg med ezetimib.	Blood lipids are measured within 1-2 months from discharge. If lipid treatment targets are not reached with a statin in the highest tolerated dose, additional therapy with ezetimibe is considered.

Om målen fortfarande inte uppnås övervägs tillägg med PCSK-9 hämmare, enligt gällande rekommendation (vid LDL ≥2,0 mmol/l).	If lipid treatment targets are still not reached, we consider additional therapy with PCSK9-inhibitor (in accordance with national prescription regulations).
Lipidstatus kontrolleras 4–6 veckor efter insättning eller dosjustering av lipidsänkande läkemedel.	Blood lipids are measured 4-6 weeks after a change has been made in lipid-lowering therapy.
Vid misstänkt FH (totalkolesterol >7,5 mmol/l och/eller LDL >5,5 mmol/l (obehandlade värden) i kombination med ärftlighet för, eller eget tidigt insjuknande i CVD, alt. beräknat DLCN score ≥6 (www.fhscore.eu)) remitteras patienten till specialistmottagning.	In the case of suspected FH (total cholesterol >7.5 mmol/L and/or LDL-C >5.5 mmol/L (measured without lipid-lowering therapy) in combination with a family history of CVD or premature CVD, or DLCN score ≥6) we refer the patient to a FH specialist clinic for further work up.
BLODSOCKER / DIABETES	*GLUCOSE METABOLISM*
Fasteblodsocker och HbA1c kontrolleras regelbundet.	Fasting glucose and HbA1c are controlled during follow-up for all patients.
Vid inkonklusiva värden för fasteblodsocker och HbA1c utförs OGTT.	When fasting glucose and/or HbA1c are inconclusive, OGTT is performed.
Vid typ-2 diabetes initieras/optimeras behandling. Våra kardiologer är bekanta med rekommenderad förstahandsbehandling och initierar denna för att minimera onödig fördröjning av behandlingsstart.	Our cardiologists initiate and optimize treatment for type-2 diabetes.
Vi samråder frikostigt med diabetologer, antingen på konsultbasis eller via integrerat samarbete.	We co-operate with diabetologists, either on a consultation basis or through integrated teamwork.
PSYKISK HÄLSA	*PSYCHOSOCIAL MANAGEMENT*
Vi frågar om psykisk hälsa, stress på arbete, hemmaplan och vad gäller relationer. Eventuella krisreaktioner efter hjärthändelsen följs upp.	Psychosocial status assessment is included in routine follow-up.
Vid psykisk ohälsa erbjuds patienter en djupare bedömning hos psykolog eller kurator.	In case of mental health issues, patients are offered a more in-depth assessment with a psychologist or social worker.
Om patienten identifieras som stressad erbjuds deltagande i stresshanteringsgrupp.	If the patient shows signs or symptoms of stress, participation in a stress management group is offered.
Vi uppmuntrar patienter med oro och nedstämdhet att delta i fysisk träning inom hjärtrehabiliteringen.	We encourage patients with anxiety and depression to participate in exercise-based CR.
Vi kopplar in kurator för praktisk hjälp och rådgivning i psykosociala frågor som rör bland annat stöd från kommun, ekonomi, boende och sysselsättning.	Vocational counselling and support are included in routine follow-up.
ÖVRIGT	*OTHER*
Patienten erhåller vid individuella möten med vården information om sin sjukdom och riskfaktorer inklusive levnadsvanor, som är anpassad till patienten i fråga. Information ges såväl muntligt som skriftligt. Digitala	During individual meetings with healthcare professionals, patients receive information about their diagnosis and risk factors, including lifestyle habits, tailored to their specific needs. Information is provided both verbally and in

informationskällor och elektroniska hjälpmedel utnyttjas.	writing, utilizing digital information sources and electronic aids if applicable.
Samtliga patienter erbjuds deltagande i interaktiv patientutbildning (till exempel Hjärtskola). Närstående erbjuds närvara vid dessa utbildningsaktiviteter.	All patients are offered participation in interactive patient education. * Relatives are offered to attend the patient education.
Vi är frikostiga med utredning av sömnapné bland patienter med kranskärlssjukdom.	We liberally refer patients with coronary artery disease for obstructive sleep apnoea work up
Sildenafil/tadalafil erbjuds till patienter med erektil dysfunktion och kronisk kranskärlssjukdom, såvida kontraindikationer inte finns.	Sildenafil/tadalafil is offered to coronary artery disease patients with erectile dysfunction if there are no contraindications to treatment.
I samband med avslut från centerbaserad hjärtrehabilitering och överlämnande till primärvården sammanfattas patientens individuella mål, mätningar, eventuella interventioner och uppnådda resultat.	In conjunction with the completion of CR programme and referral to primary care, the patient's individual goals, measurements/laboratory values, any interventions, and achieved results are summarized.

ACS, acute coronary syndrome; BMI, body mass index; CABG, coronary artery bypass grafting; CPR, cardiopulmonary resuscitation; CR, cardiac rehabilitation; CVD, cardiovascular disease; DLCN, Dutch Lipid Clinic Network; FH, familial hypercholesterolemia; HbA1c, haemoglobin A1c; LDL-C, low-density lipoprotein cholesterol; OGTT; oral glucose tolerance test; PCI, percutaneous coronary intervention.

*Variables already included in SWEDEHEART-CR. **Variables with zero variance.
