# Supplementary material for: Cohort profile: Data standards for cardiac rehabilitation structure and processes for the SWEDEHEART cardiac rehabilitation (SWEDEHEART-CR) registry
Source: PLoS One. 2023 Nov 3;18(11):e0293840. doi: 10.1371/journal.pone.0293840 (PMC10624275; doi:10.1371/journal.pone.0293840)

Journal requirements

When submitting your revision, we need you to address these additional requirements.

1. Please ensure that your manuscript meets PLOS ONE's style requirements, including those for file naming.

Answer: *We have formatted the manuscript and named all files according to PLOS ONE´s style requirements.*

2. We note that the grant information you provided in the 'Funding Information' and 'Financial Disclosure' sections do not match. When you resubmit, please ensure that you provide the correct grant numbers for the awards you received for your study in the 'Funding Information' section.

Answer: *The author(s) received no specific funding for this work. This is stated in the "Funding Information". Unfortunately, I was unable to find out how to change the "Financial Disclosure" section on the submission page. Instead, we included an updated disclosure with explanations to the change as a comment on the submission page.*

3. We note that you have indicated that data from this study are available upon request. PLOS only allows data to be available upon request if there are legal or ethical restrictions on sharing data publicly.

   In your revised cover letter, please address the following prompts:

   a. If there are ethical or legal restrictions on sharing a de-identified data set, please explain them in detail (e.g., data contain potentially sensitive information, data are owned by a third-party organization, etc.) and who has imposed them (e.g., an ethics committee). Please also provide contact information for a data access committee, ethics committee, or other institutional body to which data requests may be sent.
   b. If there are no restrictions, please upload the minimal anonymized data set necessary to replicate your study findings as either Supporting Information files or to a stable, public repository and provide us with the relevant URLs, DOIs, or accession numbers. For a list of acceptable repositories, please see http://journals.plos.org/plosone/s/data-availability#loc-recommended-repositories.

We will update your Data Availability statement on your behalf to reflect the information you provide.

Answer: *Data from the test-retest reliability testing is now included as Supporting Information (S1 Dataset). Information about retrieving patient-level data from SWEDEHEART (not used in the current manuscript) is included in the revised cover letter.*

Reviewers' comments

Reviewer #1

The manuscript describes the development of variables to audit the quality of cardiac rehabilitation and data standards in Sweden. It is well written, but some topics need to be added:

- the list of candidate variables and selected key variables (page 5)

Answer: *We thank the reviewer for this suggestion. The candidate variables have been added as Supporting Information (S1 Table). The selected key variables are listed in Table 1 (page 7-8).*

- the source for variables associated with patient outcomes

Answer: *Patient outcomes in SWEDEHEART are collected from electronic medical records, from patient interviews (self-report) or by clinical measurements. This information has been added to the manuscript* ***Baseline and follow-up data*** *section (page 10):*

Patient-level data from consecutive patient follow-up visits at the CR centre is reported at fixed time points after the MI to SWEDEHEART-CR, with data collected from electronic medical records (i.e., comorbidities, current medication, and re-admissions), self-report (i.e., symptoms, physical activity, and dietary habits), or by measurement (i.e., blood pressure, lipid levels) (Fig 2).

- it should be explained why only 4 of 6 criteria for acceptance as key variable were chosen and which criteria were used how often per candidate variable

Answer: *The Data Science Group decided upon 4 out of 6 criteria being acceptable for variables to be included. Unfortunately, we did not register which criteria were used in the selection process within the Working Group. The group came to a joint conclusion after three discussion rounds (two e-mail discussion rounds and one face-to-face meeting). This information has been added to the manuscript (page 6):*

To be accepted as key variables, candidate variables should fulfil at least 4 out of these 6 criteria. The Working Group came to a joint conclusion after three discussion rounds (two e-mail discussion rounds and one face-to-face meeting). Phrasing was further adapted after the final discussion ~~this~~ round.

- a definition of "small", "medium" and "large" CR centres is necessary (page 6)

Answer: *We thank the reviewer for pointing this out. Information about the size of centres has been added (page 7). The sentence now reads:*

"The group had a wide geographical representation and included representatives from small (less than 50 post MI patients followed per year, n=3), medium-sized (50-150 patients followed per year, n=3), and large (more than 150 patients followed per year, n=2) CR centres across the country."

- in table 1 the definition of "partly" for the parameters like human resources / centre requirements / process-based metrics is necessary and may be different for the particular parameters.

Answer: *We thank the reviewer for this valuable comment. The permissible value "Partly" (Swedish "Delvis") was included to reduce the risk of a ceiling effect. For the human resources domain, the "Partly" value enables an answer with more nuance than a binary Yes/No response if the CR team only has a part-time service from one of the professions mentioned. This mostly applies to dieticians, psychologists, and social workers. In the validity/reliability testing two sites used this permissible value for these professions. For the other variables this permissible value denotes if the routine is partly implemented/followed (i.e. using SWEDEHEART data continuously for improving quality of care) or if a part of the staff has the respective qualification (i.e. a part of the nurses in the CR team having delegation to titrate medication).*

During the development process, we received no comments from members of the SWEDEHEART-CR Working Group (n=13) or the Reference group (n=21) on this permissible value being unclear. This may be due to the word "Delvis" in Swedish being more versatile in interpretation than the English word "Partly".

We have elaborated on the choice of permissible values in the revised version of the manuscript., in the ***Development of data standards*** *section (page 5-6):*

The Data Science Group also suggested permissible values for the variables. Three values were suggested: Yes, Partly and No. The value Partly (Swedish "Delvis") was included to reduce the risk of a ceiling effect with dichotomous answers. For the human resources domain, the Partly value enables a more nuanced answer than Yes/No if the CR team only has a part-time service from one of the professions mentioned. This mostly applies to dieticians, psychologists, and social workers. For the other variables this permissible value denotes if the routine is partly implemented/followed (i.e., using registry data continuously for improving quality of care) or if a part of the staff has the respective qualification (i.e., some of the nurses in the CR team having delegation to titrate medication).

- basis of fig 3 are 8 centres (page 11), therefore it is unclear why results like ~35 (Personnel at the CR centre has training in counselling methods) are possible

Answer: *Below are the absolute numbers behind the figure. For clarification, the data supporting the figures will be added to the manuscript a Supporting information (S1 Dataset).*

	Total number of answers	Yes (n)	Yes (%)	Partly (n)	Partly (%)	No (n)	No (%)
Nurse	8	8	100%	0	0%	0	0%
Physiotherapist	8	8	100%	0	0%	0	0%
Physician	8	8	100%	0	0%	0	0%
Social worker	8	8	100%	0	0%	0	0%
Psychologist	8	0	0%	1	13%	7	88%
Dietician/nutritionist	8	5	63%	2	25%	1	13%

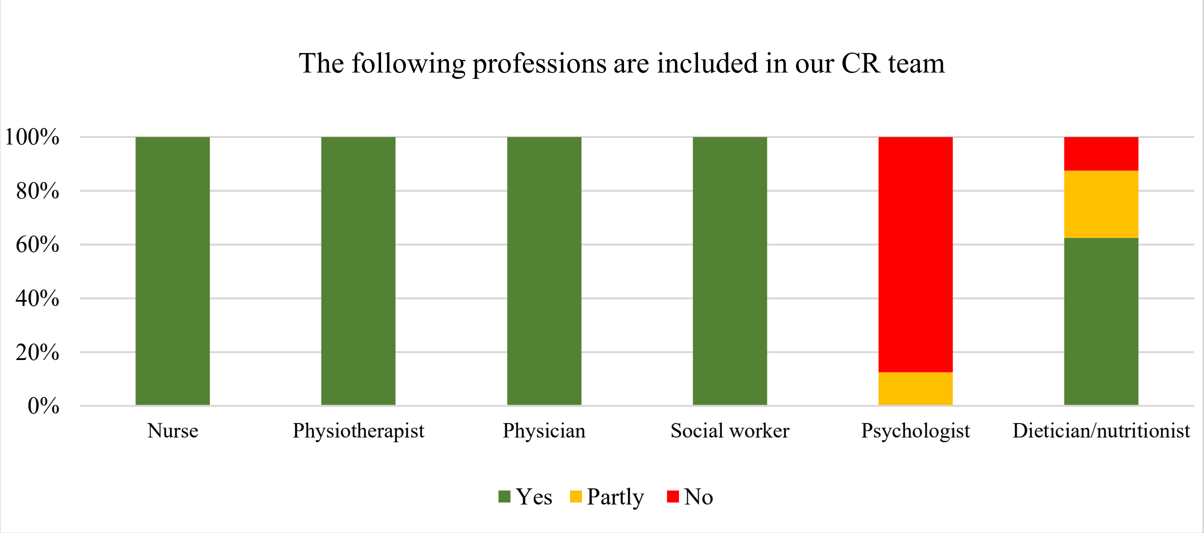

Reviewer #2

This manuscript describes data standards registry for cardiac rehab after AMI with focus on centre-level variables in addition to patient level variables. The data standards will be implemented to the excellent Swedish quality registry for cardiac diseases. The paper is well written and give useful insight on the process of choosing the variables according to current guidelines and scientific statements that can be copied by other registries in other countries. The systematic collection of the suggested data will probably improve and standardize the quality of care.

I have just some very minor comments:

I am surprised that the teams consist of psychologist (not so easily available in many places, and much of the psychological aspects could be covered by nurse and physician) and not by pharmacists, but the paper certainly does not aim to suggest how the teams are put together.

Answer: *The professions most often included in Swedish CR teams were included in the human resources domain of the data standards. According to The Perfect-CR study, a survey-based study performed by our research team in 2016, almost all (>98%) CR teams in Sweden include nurses, physiotherapists, and physicians (Michelsen H et al. Eur J Prev Cardiol. 2020 Jan;27(1):18–27). The professions most often included thereafter are social workers (approximately 90% of all teams) and dieticians (85%). Psychologists were reported to be a part of the team at approximately 15% of centres. According to the survey less than 10% reported having a pharmacist in the CR team (data not included in Michelsen´s publication).*

In table 1 I would suggest to delete the category partly for the human resources (what is a partly nurse?)

Answer: *We thank the reviewer for this valuable comment. The permissible value "Partly" (Swedish "Delvis") was included to reduce the risk of a ceiling effect. For the human resources domain, the "Partly" value enables a more nuanced response than Yes/No if the CR team only has a part-time service from one of the professions mentioned. This mostly applies to dieticians, psychologists, and social workers. During the development process, we received no comments from members of the SWEDEHEART-CR Working Group (n=13) or the Reference group (n=21) on this permissible value being unclear and in the validity/reliability testing two sites used this permissible value for these professions. Maybe this has to do with the language, the word "Delvis" being more versatile in interpretation than the English word "Partly".*

For clarification, we have elaborated on the choice of permissible values in the revised version of the manuscript., in the ***Development of data standards*** *section (page 5-6):*

The Data Science Group also suggested permissible values for the variables. Three values were suggested: Yes, Partly and No. The value Partly (Swedish "Delvis") was included to reduce the risk of a ceiling effect with dichotomous answers. For the human resources

domain, the Partly value enables a more correct answer than Yes/No if the CR team only has a part-time service from one of the professions mentioned. This mostly applies to dieticians, psychologists, and social workers. For the other variables this permissible value denotes if the routine is partly implemented/followed (i.e., using registry data continuously for improving quality of care) or if a part of the staff has the respective qualification (i.e., some of the nurses in the CR team having delegation to titrate medication).

In table 1 I would suggest to define follow-up visit (when, how many) and what is it, how comprehensive.

Answer: *We agree that defining number and timing of follow-up visits in very important. Information on the number of visits, timing and attendance is, however, already registered in SWEDEHEART-CR, these variables being described in Table 2 (page 8).*

I also think that CR programme (as for example in table 2) should be defined.

Answer: *We thank the reviewer for this comment. The definition of a comprehensive CR programme in the Introduction has been elaborated (page 3):*

Comprehensive CR is a complex multidisciplinary intervention, administered by a team and usually coordinated by a cardiologist. It comprises patient assessment, management of cardiovascular risk factors, including optimal use of cardio-protective pharmacotherapy, exercise training, behavioral modification, patient education, lifestyle and psychosocial counselling and vocational support [4].

Also, included in the manuscript is Figure 2, which describes the timeline of standard follow-up at Swedish CR centres:

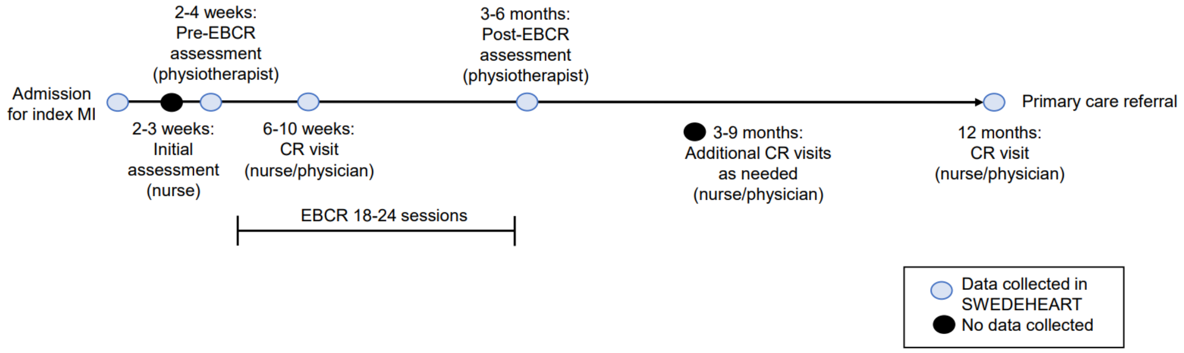

Fig 2. The basic structure of visits within cardiac rehabilitation (CR) as applied at most CR centres in Sweden. Visits at which data is collected in the SWEDEHEART registry are marked in light blue. CR, cardiac rehabilitation; EBCR, exercise-based cardiac rehabilitation; MI, myocardial infarction.

Settings and populations: Is this CR registry (and program) only for patients after an MI? What about patients with stable coronary disease but without an MI, only PCI/CABG? Do they have separate CR programs?

Answer: *The SWEDEHEART-CR registry only captures post-MI patients. Many CR centres in Sweden, however, offer a similar programme to the other ASCVD patients, as captured in the Perfect-CR survey and shown in the graph below (unpublished data).*

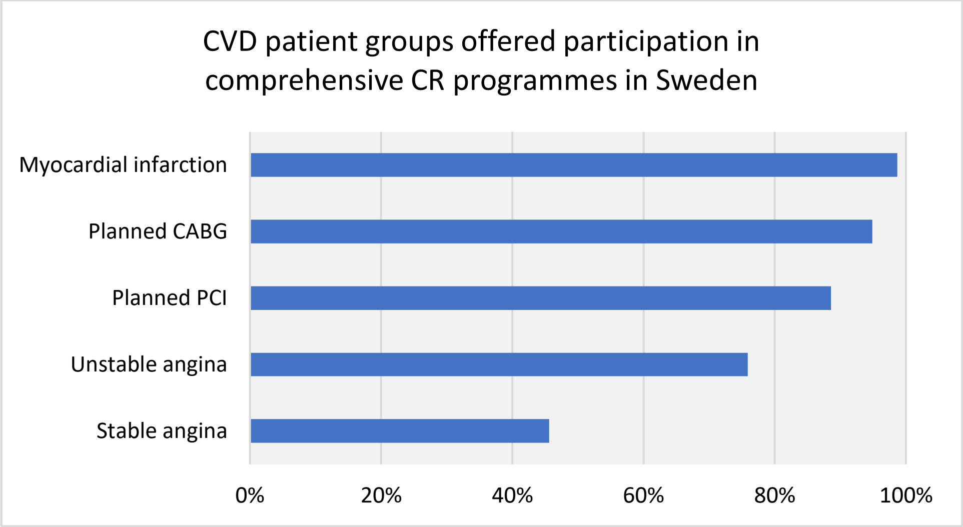

Supplement: Attachment — Submitted filename: Response_to_reviewers.pdf [file pone.0293840.s003.pdf]
